# Targeting tumor-associated macrophages to combat pancreatic cancer

**DOI:** 10.18632/oncotarget.9383

**Published:** 2016-05-15

**Authors:** Ran Cui, Wen Yue, Edmund C. Lattime, Mark N. Stein, Qing Xu, Xiang-Lin Tan

**Affiliations:** ^1^ Department of Oncology, Shanghai Tenth People&rsquo;s Hospital, Tongji University, School of Medicine, Shanghai, P. R. China; ^2^ Department of Oncology, Shanghai Medical College, Fudan University, Shanghai, P. R. China; ^3^ Rutgers Cancer Institute of New Jersey, Rutgers, The State University of New Jersey, New Brunswick, NJ, USA; ^4^ Department of Epidemiology, School of Public Health, Rutgers, The State University of New Jersey, Piscataway, NJ, USA

**Keywords:** tumor-associated macrophages, tumor microenvironment, pancreatic cancer, chemoprevention

## Abstract

The tumor microenvironment is replete with cells that evolve with and provide support to tumor cells during the transition to malignancy. The hijacking of the immune system in the pancreatic tumor microenvironment is suggested to contribute to the failure to date to produce significant improvements in pancreatic cancer survival by various chemotherapeutics. Regulatory T cells, myeloid derived suppressor cells, and fibroblasts, all of which constitute a complex ecology microenvironment, can suppress CD8+ T cells and NK cells, thus inhibiting effector immune responses. Tumor-associated macrophages (TAM) are versatile immune cells that can express different functional programs in response to stimuli in tumor microenvironment at different stages of pancreatic cancer development. TAM have been implicated in suppression of anti-tumorigenic immune responses, promotion of cancer cell proliferation, stimulation of tumor angiogenesis and extracellular matrix breakdown, and subsequent enhancement of tumor invasion and metastasis. Many emerging agents that have demonstrated efficacy in combating other types of tumors via modulation of macrophages in tumor microenvironments are, however, only marginally studied for pancreatic cancer prevention and treatment. A better understanding of the paradoxical roles of TAM in pancreatic cancer may pave the way to novel preventive and therapeutic approaches. Here we give an overview of the recruitment and differentiation of macrophages, TAM and pancreatic cancer progression and prognosis, as well as the potential preventive and therapeutic targets that interact with TAM for pancreatic cancer prevention and treatment.

## INTRODUCTION

Pancreatic ductal adenocarcinoma (PDA), which accounts for more than 90% pancreatic cancer cases, is one of the most aggressive malignancies, with poor response of tumors to conventional therapeutic intervention chemotherapy and radiotherapy [1]. As PDA predominantly develops without early symptoms, the majority (85-90%) of PDA patients initially present with locally advanced and metastatic disease for which therapeutic options are very limited, in line with low overall 5-year survival rate of 6% [2]. By 2020, PDA is anticipated to become the second leading cause of cancer death in the United State, overtaking deaths from breast and colon cancers [3]. There is an urgent and unmet medical need to discover novel and more effective therapeutic and preventive strategies for this lethal disease.

Some key characteristics of PDA, such as the desmoplasia (tumor stroma), restricted vasculature and hypoxic environment may prevent the delivery of chemotherapy to the tumor thereby explaining the limited benefits observed to-date. PDA is one of the most stroma-rich cancers, which in some cases up to 80% of the tumor mass are made up by stromal tissue [4]. In PDA, the tumor stroma comprises abundant fibrotic tissue composed of infiltrating immune cells [being most prominent for immunosuppressive leukocytes, including macrophages, myeloid-derived suppressor cells (MDSC), and regulatory T cells (Treg)], pancreatic stellate cells, vascular cells, fibroblasts, myofibroblasts and other extracellular matrix components [5]. Although each component of the stromal compartment was described as promoting PDA progression, this review will focus on the recent findings of tumor promoting functions of macrophages.

In fact, immune cells are one of the most important supportive cells in PDA progression, and one of the reasons for the resilience of PDA towards intensive treatment is that the cancer is capable of hijacking the immune system [6]. Macrophages, an essential component of the innate immune system in humans, represent the major immune cell type present in the PDA tumor microenvironment, where they are commonly termed tumor-associated macrophages (TAM) [7]. TAM, derived from circulating monocytes, are the main population of inflammatory cells in the solid tumors and key regulators of the link between inflammation and various types of cancer [8-11]. TAM can generally promote cancer cell proliferation, stimulate tumor angiogenesis and extracellular matrix breakdown, and subsequently enhance tumor invasion and metastasis [12]. TAM are also immunosuppressive, preventing tumor cells attacked by natural killer and T cells during tumor progression and after recovery from chemo- or immunotherapy [13]. Given that the limited benefits have been observed for the current conventional therapeutic intervention which targets cancer cells itself, targeting TAM provides an opportunity for the prevention and treatment of pancreatic cancer. In this review, we give an overview of the recruitment and differentiation of macrophages, TAM and PDA progression and prognosis, as well as the potential preventive and therapeutic targets that interact with TAM for pancreatic cancer prevention and treatment.

## TAM AND PROGNOSIS IN PANCREATIC CANCER

**Figure 1:**
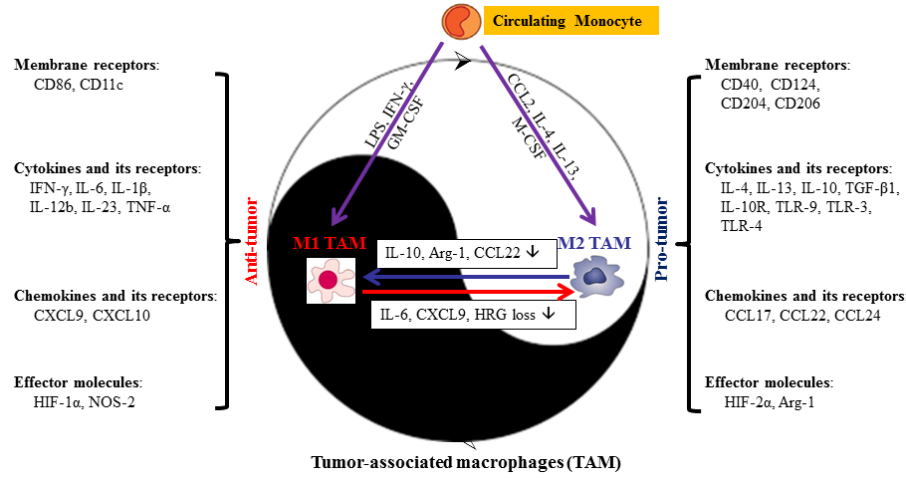
Dual role of macrophages in the response to stimuli in tumor microenvironment Briefly, stimuli that polarize macrophages are divided into membrane receptors, cytokines and its receptors, chemokines and its receptors, and effector molecules. The majority of molecules listed above are secreted by both M1- and M2-TAM. However, the phenotype of TAM may vary during different stages of tumor progression, resulting in differential stimuli density in M1- or M2-TAM. Arg, arginase; CCL, C-C chemokine ligand; GM-CSF, granulocyte macrophage colony-stimulating factor; HIF, hypoxia-inducible factor; HRG, histidine-rich glycoprotein; IFN-γ, interferon-γ; IL, interleukin; M-CSF, macrophage colony-stimulating factor; MR, mannose receptor; NOS, nitric oxide synthase; TAM, tumor-associated macrophages; TGF, transforming growth factor; TLR, toll-like receptor; TNF, tumor necrosis factor.

Macrophages shift their functional phenotype in response to various microenvironmental stimuli such as IL-10, TGF-α and other cytokines. Based on their function, macrophages are divided broadly into two categories: M1 (pro-inflammatory or ‘classically activated’ macrophages) and M2 (anti-inflammatory or ‘alternatively activated’ macrophages) (Figure 1). The macrophage phenotype transition from M1 to M2 is believed to be a remarkable event in tumor initiation and progression. Macrophages in tumors (e.g. TAM) closely resemble the M2-polarized macrophages and are critical modulators of the tumor microenvironment. TAM promote tumor progression by suppressing an anti-tumor immune response, stimulating vascularization, invasion, metastasis, and increasing the tumorigenicity of cancer stem cells [14-18]. Approximately 80% of tumors have shown a positive correlation between the poor prognosis and TAM, while only tumors less than 10% of TAM density demonstrated good prognosis [19]. M2-polarized macrophages (identified by CD163 immunopositivity) were significantly more abundant in primary PDA samples, compared to the paired adjacent normal tissues and those diagnosed as chronic pancreatitis [20]. The presence of M2 polarized TAM in the stroma are strongly correlated with the tumors located in the tail and body of the pancreas [20], and higher counts of M2-polarized TAM were associated with increased risk of lymph node metastasis, neural invasion, chemoresistance, and hence the worse prognosis and survival in PDA [21-23].

However, recent accumulating evidence shows that TAM often share features of both M1 and M2 rather than could be classified into two classes, and the phenotype of TAM may vary during different stages of tumor progression [24]. For instance, macrophages may function with an M1-like phenotype during the initiation of tumor, and switch to an M2-like phenotype when the tumor begins to invade and metastasize [27, 28]. In pancreatic cancer, TAM also exhibit characteristics of both pro- and anti-inflammation properties and contribute to epithelial-mesenchymal-transition (EMT) [25, 26], reflecting the plasticity and heterogeneity of TAM. In addition, recent reports suggested that cancer treatment may also modulate macrophages phenotype, while the regional microenvironment, which is featured by dense stroma infiltration, generally plays a decisive role in recruiting monocytes and modulating macrophage phenotype. Low-dose irradiation might redirect macrophage differentiation from a tumor-promoting/immunosuppressive state to an anti-tumor immunostimulatory phenotype, indicating a possibility of shifting between M1 and M2 [29]. Neoadjuvant cyclophosphamide was also found to decrease density of CD206^+^ and IL-10^+^ TAM (M2-like phenotype) at the PDA-stroma interface, moreover, *in vitro*, gemcitabine-treated macrophages became tumoricidal, inhibiting their protumoural effect and switching to an antitumour phenotype [30]. The plasticity and heterogeneity of TAM in pancreatic cancer may be one of the major reasons for the poor prognosis of cancer patients and presents a significant challenge for targeting TAM in pancreatic cancer.

## RECRUITMENT AND CHARACTERISTICS OF TAM IN PANCREATIC CANCER

### Macrophage infiltration and origins of TAM

In tumor microenvironment, TAM originate from the circulating monocyte, and acquire either a classical M1 or alternative M2 phenotype depending on microenvironmental stimuli. The recruitment of macrophages into tumors is primarily regulated by cytokines, chemokines, and growth factors that are derived from tumor and stromal cells in the tumor microenvironment. One of these chemokines, tumor-derived C-C chemokine ligand 2 (CCL2) acts as a potent factor for Th2 polarization and shifts monocytes toward the M2-polarized macrophages (Figure 1). CCL2 acting via its receptor C-C chemokine receptor 2 (CCR2) is a direct mediator of monocyte recruitment to the primary tumor and to metastases in the Polyoma Middle T oncoprotein (PyMT) model [31, 32]. Under physiologic conditions, the CCL2/CCR2 chemokine axis plays an important role in the mobilization of inflammatory monocytes from the bone marrow to the blood [36]. Studies had proposed a dual role of CCL2 on tumor growth, and CCL2 can be protective in some tumor models but destructive in others [37, 38]. Overexpression of CCL2 by tumor cells can lead to their destruction by an infiltrate of activated mononuclear cells and may indicate a more favorable prognosis.

Pancreatic cancer is rich in stroma, which facilitate the secretion of CCL2 [41, 42]. It has been reported that the overproduction of CCL2 is closely related to macrophage infiltration in pancreatic cancer [33-35]. The potential contribution of CCL2 to pancreatic cancer progression is of major interest because CCL2 production correlated with the levels of macrophage content in transplanted tumors *in vivo* [43]. Sanford *et al*. found that an increased ratio of inflammatory monocytes in the blood versus the bone marrow is a novel predictor of decreased pancreatic cancer patient survival following tumor resection, and patients with high CCL2 expression/low CD8 T cell infiltrate have significantly decreased survival [39]. Monti *et al*. [44] also showed that pancreatic cancer patients with high circulating CCL2 have higher numbers of CD68+ tumor-infiltrating macrophages, and circulating levels of CCL2 are inversely correlated with Ki-67, a well-established marker of tumor cell proliferation. However, low level CCL2 secretion, with physiological accumulation of TAM, promoted tumor formation.

CCL5 produced by naive T cells and tumor cells also contributes to monocyte migration into tumor sites [45]. While the presence of other CC chemokines including CCL3, CCL4, CCL18 and CCL22 and some CXC chemokines, in particular CXCL8 [46], has been correlated with the presence of macrophages, it still remains to be determined whether they play a role in the recruitment or in the maintenance of the TAM in tumor tissue [47]. Interestingly, CCL5 stimulates human monocytes to express CCL2, CCL3, CCL4 and CXCL8, all of them chemoattractants for myeloid cells [48].

Along with the chemokines, several cytokines such as macrophage colony-stimulating factor (M-CSF or CSF-1) and endothelial monocyte-activating polypetiptide II (EMAPII) have been implicated in the recruitment of monocytes into tumors [53]. While high levels of CSF-1 were often observed in luminal breast cancer cells, CSF-1 is not frequently overexpressed by pancreatic cancer cells [54]. By contrast, granulocyte macrophage (GM)-CSF (CSF-2) is commonly expressed in pancreatic cancer cells and has been linked to the generation of MDSC with immunosuppressive features [55]. Additionally, certain growth factors including vascular endothelial growth factor (VEGF) [56], platelet-derived growth factor (PDGF) [57] have also been reported to promote monocyte/macrophage recruitment. However, the anti-angiogenic drug sunitinib, which targets VEGF and PDGF receptor signaling, did not inhibit PDA progression in a mouse model, although it showed temporal blockage of tumor growth in several tumor animal models [58].

### Macrophage polarization

A network of signaling molecules, transcription factors, epigenetic mechanisms and posttranscriptional regulators participate in directing macrophage polarization. The detailed molecular determinants of macrophage polarization have been outlined and reviewed [24]. In brief, stimulation with lipopolysaccharide (LPS) and interferon-γ (IFN-γ) leads to M1 activation and the secretion of IL-1α, IL-6 and tumor necrosis factor α (TNF-α) along with the expression of leukocyte antigen (HLA)-DR^high^, CD11c, NOS-2, IL-12b, and IL-23. M2 macrophages, in contrast, are activated by IL-4 and IL-13, secrete IL-10 and expression makers such as HLA-DR^low^, CD124 (IL-4Rα), CD204 (SR-A), CD206 (mannose receptor), arginase 1 (Arg-1), and stabilin-1 [59, 60] (Figure 1). Notably, M-CSF-driven macrophage differentiation leads to the expression of a substantial part of M2 transcriptome, while GM-CSF rather induces an M1-type of activation [47, 61]. Interestingly, the hypoxia-inducible factors (HIF)-1α and HIF-2α are expressed differentially in M1- and M2-polarized macrophages [62] and regulate inducible NOS-2 (M1) and Arg-1 (M2), respectively (Figure 1), suggesting that tumor hypoxia may mediate the transition of M1 to M2.

Recently, several regulators have been suggested to mediate the polarization of macrophage in pancreatic cancer. Histidine-rich glycoprotein (HRG), a multidomain plasma protein synthesized by hepatocytes, has been reported to skew TAM polarization away from the M2- to a tumor-inhibiting M1-phenotype in orthotopic Panc02 tumor models [63]. Heparanase, which is an endoglycosidase that cleaves heparan sulfate (HS) glycosaminoglycans, can also guide cancer-promoting action of TAM by augmenting STAT3 signaling *in vitro* and in a mouse model of heparanase-overexpressing pancreatic carcinoma [64]. Reg3α, a small secretory protein belonging to the calcium-dependent lectin superfamily, also contribute to M2-polarized phenotype through the activation of STAT3 pathway in an orthotopic mouse model of pancreatic cancer [65]. Additionally, the homeobox transcription factor CUX1 has been shown to antagonize M1 polarization by negatively interfering with NF-κB signaling *in vitro* and in *LSL-Kras^G12D/+^*; *LSL-Trp53^R172H/+^*; *Pdx-1-Cre* mice [66]. These studies highlight a potential therapeutic opportunity in which re-educating TAM might have beneficial anti-tumorigenic effects on pancreatic cancer.

## TAM AND TUMOR PROGRESSION IN PANCREATIC CANCER

### TAM and regulation of angiogenesis and hypoxia

**Figure 2:**
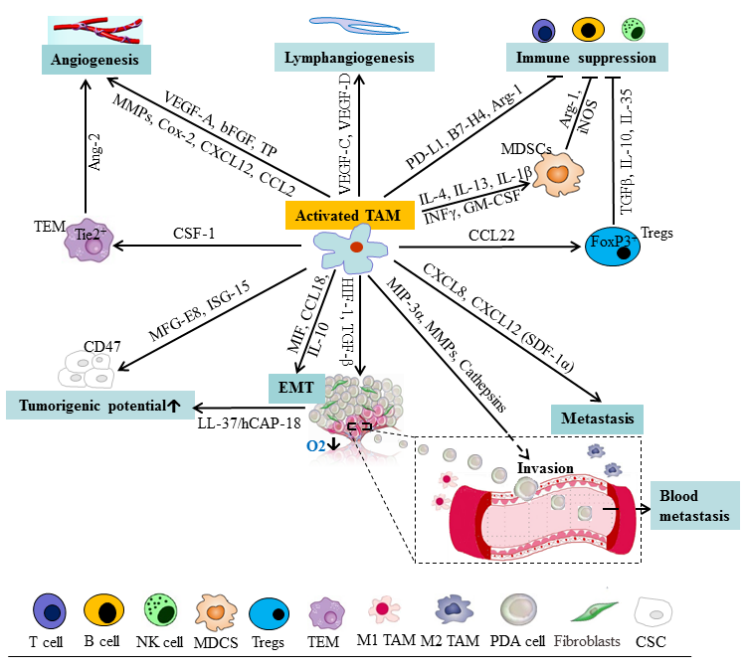
Schematic representation of cells and mediators influencing the function of TAM and tumor progression in pancreatic cancer TAM can release a panel of mediators facilitating lymphangiogenesis, angiogenesis, EMT, immune suppression, and tumorigenicity of CSC, which provide a permissive environment for pancreatic tumor progression. Ang-2, angiopoietin-2; CSC, cancer stem cells; EMT, epithelial-mesenchymal transition; FGF, fibroblast growth factor; ISG-15, interferon-stimulated gene 15; MDSC, myeloid-derived suppressor cells; MFG-E8, milk-fat globule-epidermal growth factor-VIII; MIF, migration inhibitory factor; MMP, matrix metalloproteinases; NK, nature killer; NOS, nitric oxide synthase; PD, programmed death; PDA, pancreatic ductal adenocarcinoma; TAM, tumor-associated macrophage; TEM, Tie2-expressing monocytes; TGF, transforming growth factor; TP, thymidine phosphorylase; Treg, regulatory T cells.

Angiogenesis sustained by mediators produced by tumor and stromal cells provides oxygen and nutrients to allow cancer cells to multiply, invade nearby tissue, and metastasize. TAM can accelerate vessel growth by releasing a panel of pro-angiogenic factors, such as vascular endothelial growth factor A (VEGF-A), TNF-α, basic fibroblast growth factor (bFGF), and the angiogenic factor thymidine phosphorylase (TP) (Figure 2). Of these factors, VEGF-A is the best characterized one and is recognized as a major pro-angiogenic cytokine released by TAM [67]. VEGF-A stimulates angiogenesis by promoting endothelial cell migration and proliferation via binding with its corresponding tyrosine kinase receptors, VEGFR-1 and VEGFR-2 [68]. Additionally, TAM also involved in angiogenic processes by producing several angiogenesis-modulating enzymes such as MMP-2, MMP-7, MMP-9, MMP-12 and cyclooxygenase-2 (Cox-2), and chemokines such as CXCL12, CCL2, CXCL8, CXCL1, CXCL13 and CCL5 (Figure 2). TAM can express proteases to release a number of pro-angiogenic molecules bound to heparan sulfate in proteoglycans, and fragment of fibrin and collagen, which facilitate angiogenesis [71]. Among these, MMP-9 [72], urokinase plasminogen activator (uPA) and receptor [73-76] are the prominent ones which promote tumor directed angiogenesis.

Paradoxically, unlike the majority of other tumor types which are clearly dependent on angiogenesis, PDA is characterized by hypovascularity [77], and PDA tumor samples (both in mice and human) show substantially lower microvessel densities than those of the normal pancreas [78]. However, mechanisms behind these histopathological features have not been fully elucidated. Discouraging results of antiangiogenic therapies in clinical and preclinical studies add to mounting evidence of angiogenetic independence and dominance of tumor driven angiostasis of PDA, suggesting that tumor angiogenesis might affect PDA progression to a lesser extent than in other cancers [79, 80]. Despite this, the overexpression of VEGF in PDA has been reported to be closely related to a high microvessel density, disease progression, increased risk of metastatic spread, and a poor prognosis [70]. VEGFR-2 is essential for VEGF-stimulated migration of TAM, and selective inhibition of VEGFR-2 reduces recruitment of macrophages into orthotopic pancreatic tumors in mice [69]. Therefore, alternative approaches targeting the PDA vasculature remain attractive and potentially feasible. It is conceivable and important to figure out whether TAM confer PDA to desmoplastic reaction and hypovascularity.

In addition, pancreatic tumors are frequently marked by regions of hypoxia, as rapidly dividing malignant cells outpace the capacity of the established vasculature to deliver oxygen and nutrients. In this context, hypoxia is associated with restrained proliferation, differentiation, necrosis or apoptosis, and it can also lead to the development of an aggressive phenotype [81] (Figure 2). TAM have localized in the hypoxic regions into the tumor microenvironment which promoted the expression of HIF-1 and HIF-2. HIF-1α is a major regulator of the production of VEGF-A in TAM. HIF-1α induces VEGF expression and recruits CD45+ monocytic cells which in turn enhance the mobilization of VEGF from the extracellular matrix. This translocation makes VEGF available for its receptor VEGFR-2 and may cause vascular remodeling and neovascularization [82]. Knockout of HIF-1α in TAM enhances M2 polarization and attenuates their pro-angiogenic responses [83].

TGF-α is another common TAM-produced cytokine that can induce VEGF-A through HIF-1 pathway. TGF-α is the prototype of a large superfamily of secreted signaling polypeptides [including TGF-α, bone morphogenic proteins (BMP), and activin/inhibin], with diverse functions in the development and pathogenesis of a variety of diseases [84, 85]. Jeon *et al*. found that TGF-α induced the transcription and secretion of VEGF through the synergistic action of HIF-1α and Smad3/4 in macrophages [86]. Recently, an intrinsic angiogenesis inhibitor, vasohibin-1, has been shown to be regulated by the TGF-α/BMP signaling between TAM and pancreatic cancer cells [87].

Apart from VEGF, HIF-1α, and TGF-α, angiopoietin-2 (Ang-2), a ligand of the Tie2 tyrosine kinase receptor, has also been reported to be involved in TAM-regulated angiogenesis in tumor microenvironment (Figure 2). Targeting Ang-2 or Tie2 releases the macrophage-vessel association and inhibits angiogenesis in RIP1-Tag2 transgenic pancreatic islet adenocarcinomas mouse model [88]. Tie2 is primarily expressed on endothelial cells, but also expressed on some monocytes/macrophages. Tie2-expressing monocytes/macrophages (TEM) are characterized by the expression of Tie2 receptor and accumulate in hypoxic areas of tumors in response to Ang-2 produced at high levels by hypoxic vascular cells. TEM with pro-angiogenic and pro-tumorigenic activity play a pivotal role in the angiogenic switch [90], and have been identified in several mouse tumor models, including RIP1-Tag2 transgenic pancreatic islet adenocarcinomas [91, 92]. Selective depletion of TEM in RIP1-Tag2 transgenic mice during early stages of tumor development prevents tumor neovascularization and inhibits tumor growth [91]. It has also been suggested that hypoxia up-regulates Tie2 expression on TEM and, together with Ang-2, down-regulates their antitumor functions [93]. Interestingly, CSF-1 upregulates Tie2 on TAM, indicating a link between CSF-1, Tie2^+^ macrophages, and the induction of the angiogenic switch [94]. Given their tumor-homing ability and selectivity, TEM may be used as surrogate makers to monitor angiogenesis *in vivo* or as gene delivery vehicles to restrict antiangiogenic or antitumor gene expression to the tumor site [93].

In addition, TAM are involved in lymphangiogenesis, a process mediated by a number of factors including VEGF-C and VEGF-D via VEGFR3. It has been reported that VEGF-C expressing TAM secreted lymphatic endothelial growth factors which are related to peritumoral lymphangiogenesis in human cervical cancers [95, 96]. VEGF-A, an essential hemangiogenic factor, has also been shown to increase lymphangiogenesis via recruitment of monocytes/macrophages using a mouse model in which both lymphangiogenesis and hemangiogenesis are induced in the normally avascular cornea [97].

### TAM and immunosuppression

TAM convey immunosuppressive activity through secretion of immunosuppressive cytokines, chemokines, and proteases, such as TGF-α, IL-10, CCL17, CCL22, and Arg-1. The role of TAM-derived cytokines, chemokines, and proteases in immunosuppression has been reviewed previously [98]. TAM may also promote apoptosis of T cells by expressing the inhibitory B7 family molecules, such as B7 homolog 1 (B7-H1) or programmed death-ligand 1 (PD-L1) on their cell surface. Here, we are focusing on the roles of TAM in pancreatic cancer immunosuppression.

Pancreatic tumor microenvironment is immunosuppressive, which limit the activation of immune responses and inhibit the function of activated immune effectors [104]. Of all the different types of immune cells, Treg, which defined as CD4^+^CD25^+^FoxP3^+^ cells, have gotten the most attention in pancreatic tumor immunology research. The mechanism of inhibitory activity of Treg on immune suppression remains to be completed understood, but involves the production of inhibitory cytokines, such as TGFα, IL-10 and IL-35 (Figure 2). Treg produce a local immunosuppressive environment ideal for tumor growth. The selective recruitment of Treg by tumor-derived endothelial cells is in part response by Treg to chemokines such as CCL-22, which is expressed by TAM. FoxP3 transcription factor, which is crucial for Tregs survival and function, expressed in PDA cells and inhibit naive T cell proliferation, but not activation. In the mutant *k-ras* model of pancreatic cancer, Tregs are present in increased amounts within the tumor microenvironment early in disease progression [106]. Patients with pancreatic cancer have increased numbers of Tregs both in the circulation and at the tumor site, and low Tregs percentage in the circulation one year post resection correlates with improved survival [105].

Other cells and tumor molecules effecting immune defects in pancreatic cancer include MDSC, TGF-α, mucins, COX-2-derived PGE-2, MHC I-related chain A/B molecules (MICA/B), PD-L1/PD-H1 and PD-L2/PD-DC. The expansion and activation of MDSCs is regulated by several factors (e.g. IL-4, IL-13, IL-1α, INFγ and GM-CSF) that are released by activated macrophages and T cells, tumor cells, tumor stromal cells, and by pathogen-infected cells [107]. Immature mononuclear cells within the MDSC population are precursors for immunosuppressive TAM, which have an ability to induce energy or T-cell tolerance due to downregulation of HLA-DR molecules on TAM [108]. MDSC express high levels of both Arg-1 and iNOS, which play a direct role in the inhibition of CD8^+^ T-cell function [109]. In contrast to MDSCs, TAM upregulate the expression of either Arg-1 or iNOS, depending on the nature of the tumor microenvironment, but not of both proteins [107]. In addition, TGF-α, which secreted by the tumor cells and the MDSC population, has immunosuppressive properties that include inhibition of T cell activation, proliferation and differentiation into CTL or Th cell subsets, as well as drives the development of FoxP3^+^, CD4^+^, CD25^+^ Treg which suppress antitumor immunity [110, 111]. In a genetically defined PDA mouse model, Clark and colleagues [112] observed a prominent leukocytic infiltration even around the lowest grade pre-invasive lesions, while immunosuppressive cells, including TAM, MDSC, and Treg, dominated the early response and persisted through invasive pancreatic cancer.

It is important to note that TAM, MDSC, and Treg express high levels of the ligand receptors for immune-checkpoint PD-1 and cytotoxic T-lymphocyte-associated antigen 4 (CTLA-4) that upon activation suppress cytotoxic functions of T cells, NK T cells and NK cells. Since pancreatic tumors are highly infiltrated with these cellular suppressors of effector immune response, blockade of the PD-1 and CTLA-4 pathway may enhance antitumor immune response by diminishing the number and/or suppressive activity of these three intratumoral suppressor cells. However, single-agent checkpoint inhibitors that alter immune suppressive signals in other human cancer such as CTLA-4, PD-1 and its ligand PD-L1, have failed to demonstrated objective response when given as single agents to PDA patients [113]. A possible explanation for the therapeutic failure of CTLA-4, PD-1 or PD-L1 blockade therapy in PDA is the lack of a nature infiltration of effector immune cells in the majority of PDA. A potential strategy to activate effector T-cell trafficking into the tumor microenvironment is vaccine-based immunotherapy. Given that cancer vaccine-based immunotherapy may overcome the resistance of PDA to immune checkpoint inhibitors, while immune checkpoint inhibitors may enhance the efficacy of the cancer-vaccine therapies, combining vaccine therapy with dual blockade of CTLA-4 and PD-1 might be an attractive approach, although the autoimmune toxicities can be concern.

### TAM and EMT

Epithelial-mesenchymal transition (EMT) is an important biological process by which epithelial tumor cells lose epithelial features and gain mesenchymal phenotypes in the progression of primary tumors toward metastasis [114]. E-cadherin, an epithelial cell marker, diminishes during EMT, while mesenchymal cell markers are upregulated. Interestingly, the expression of E-cadherin can also be repressed by the mesenchymal markers, Snail and Slug. These markers have been identified in tumor tissue following pancreatic resection [115]. Pancreatic cancer cell lines (PANC-1 and BxPC-3) co-cultured with M2-polarized TAM could upregulate mesenchymal markers vimentin and Snail at the mRNA and protein levels, augment the proteolytic activity of metalloproteinases MMP-2 and MMP-9, and decrease the expression of the epithelial marker E-cadherin [116].

TAM have been found to enhance EMT in various cancer types. For example, TAM induced EMT in lung cancer cells through paracrine TGF-α signaling and consecutive activation of the α-catenin pathway [117]. In hepatocellular carcinoma, TAM may induce EMT via the IL-8 activated JAK2/STAT3/Snail pathway [118]. Recently, Su *et al*. reported that TAM in breast cancer induced EMT via CCL-18-induced NF-κB activation to form a positive feedback loop between mesenchymal-like cancer cells and macrophages [119]. In pancreatic cancer, both M1 and M2 macrophages were equally capable of promoting EMT in malignant and premalignant pancreatic ductal epithelial cells [26]. Several cytokines, such as interleukin-10 (IL-10) [116], macrophage migration inhibitory factor (MIF) [120], and CCL-18 [121], have been shown to induce EMT in pancreatic cancer cells (Figure 2). It is possible that these inflammatory factors released by TAM may work coordinately to provide a permissive environment for pancreatic tumor progression.

### TAM and pancreatic cancer stem cells

Increasing evidence indicates that cancer stem cells (CSC) play a major role in the development and metastatic progression of PDA [122, 123]. TAM have recently been found to regulate the self-renewal, tumorigenic and metastatic potential of CSC in various cancer types [18]. TAM can also interact with and promote the tumorigenicity of CSC via production of the milk-fat globule-epidermal growth factor-VIII (MFG-E8), a major regulator of CSC activities, which activates STAT3 and sonic hedgehog pathways in CSC [18]. In pancreatic cancer, inhibition of macrophage recruitment by targeting either the CSF-1 receptor (CSF-1R) (PLX6134, PLX3397) or CCR-2 (PF-04136309) resulted in a significant reduction of pancreatic cancer cells expressing high levels of the CSC marker ALDH [124]. Interestingly, TAM can directly enhance the tumor-initiating capacity of pancreatic cancer cells by activating the transcription factor STAT3, thereby facilitating macrophage-mediated suppression of CD8^+^ T lymphocytes [124]. Recently, Sainz *et al*. showed that interferon-stimulated gene 15 (ISG-15), which is an IFN-α/α-inducible ubiquitin-like modifier, was expressed and secreted by TAM in response to IFN-α produced by PDA cells. ISG-15 then reinforced the self-renewal, invasive capacity, and tumorigenic potential of CSC [125] (Figure 2).

In addition, CD47, a ligand to signal regulatory protein α (SIRPα) on macrophages, is highly expressed on pancreatic CSC, and the high expression of CD47 in CSC could communicate to the SIRPα and result in the inhibition of phagocytosis by macrophages [126]. A recent *in vivo* and *in vitro* study [127] revealed that anti-CD47 antibody not only enables phagocytosis of these cells by macrophages, but also directly induces apoptosis of PDA CSC, while exerting no effect on non-malignant cells.

Interestingly, an antimicrobial peptide, LL-37/hCAP-18 secreted by TAM in response to CSC-secreted Nodal/Activin A is strongly expressed in the stroma of PDA [128]. LL-37 could increase pluripotency-associated gene expression, self-renewal, invasion and tumourigenicity of CSC via formyl peptide receptor 2 (FPR2)- and P2X purinoceptor 7 receptor (P2×7R)-dependent mechanisms (Figure 2). In a *k-ras*-driven pancreatic tumourigenesis mouse model, pharmacologically inhibition of FPR2 and P2×7R could effectively inhibit tumor formation [128]. Taken together, these studies supported that TAM play a critical role in the induction, maintenance, and expansion of CSC in the tumor microenvironment. Targeting pancreatic CSC in combination with interrupting the interplay between pancreatic CSC and TAM may provide more effective clinical treatments for pancreatic cancer.

### TAM and tumor invasion and metastasis in pancreatic cancer

TAM promote tumor metastasis by enhancing the invasion of malignant cells and also by influencing tumor microenvironment via secreting matrix proteins and several proteases such as serine proteases, MMP and cathepsins, which act on cell-cell junctions, modify the ECM composition, and promote basal membrane disruption. In human pancreatic cancer, the macrophage inflammatory protein-3 alpha (MIP-3α) expressed by TAM and pancreatic cancer cells has been implicated as a regulator of tumor cell invasion [129] (Figure 2). MIP-3α induced MMP9 expression of pancreatic cells through its receptor CCR6 and consequently increased pancreatic cancer cell invasion in collagen Type IV [130, 131]. In a pancreatic tumor mouse model, inhibition of macrophage recruitment using the CSF-1R inhibitors reduced metastatic spreading to liver [124], while increased macrophage conversion towards to the M2 phenotype by the loss of histidine-rich glycoprotein (HRG) increased metastatic spreading [17]. In addition, CXCL8/IL-8 and CXCL12/stromal cell-derived factor-1α (SDF-1α) can co-operatively promote migration/invasion and angiogenesis in pancreatic cancer, [132] indicating that CXCR2 and CXCR4, corresponding receptors for CXCL8 and CXCL12 respectively, are potential anti-angiogenic and anti-metastatic therapeutic targets in pancreatic cancer.

Macrophage can also promote pancreatic tumor metastasis through their ability to promote lymph angiogenesis either by the secretion of the lymphatic endothelial growth factor VEGF-C [133] or their direct integration into tumor associated lymphatic vessels [134]. It has been reported that M2-polarized TAM infiltration of regional lymph nodes was significantly associated with nodal lymphangiogenesis [135], and M2-polarized TAM identified by CD163 and CD206 in the invasion front of human pancreatic cancer were associated with a poor prognosis due to accelerated lymphatic metastasis [21]. Therefore, inhibition of the functional interaction between M2-polarized TAM and lymphangiogenesis may suppress tumor invasion and metastasis in pancreatic cancer.

Additionally, endoneurial macrophages that lie around nerves may also foster the invasion of pancreatic cancer cells along the nerves through secreting high levels of glial-derived neurotrophic factor (GDNF), thus inducing the activation of the GNDF receptor GFRα1 and its co-receptor RET [136]. RET is involved in tumorigenesis through multiple distinct signaling pathways such as RAS-MAPK, PI3K-AKT, and JAK-STAT3 [137-139], and has been shown to be expressed in 50-65% of PaCA and to be correlated to advanced metastatic status [139-141]. Activated RET in PaCA cells induces cell migration towards nerves in both *in vitro* and *in vivo* models of perineural invasion [136, 142, 143]. Targeting RET might be an attractive approach for prevention and treatment of PDA metastasis.

## TARGETING MACROPHAGE IN PANCREATIC CANCER

### Inhibiting monocyte/macrophage recruitment

**Figure 3:**
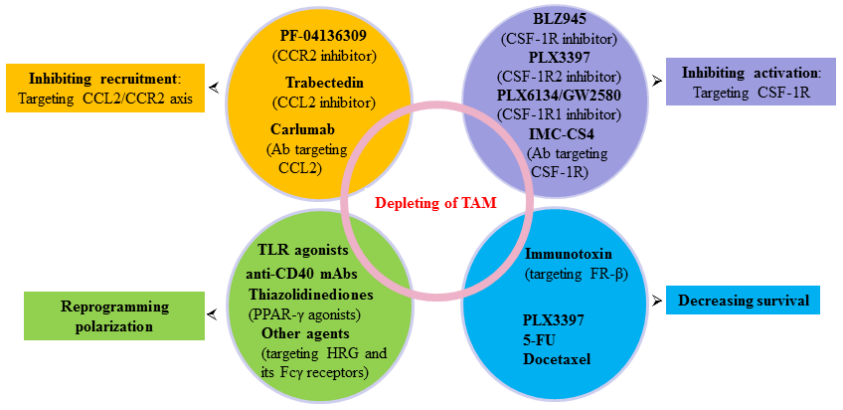
Agents targeting TAM in pancreatic cancer Multiple agents and strategies to target TAM as indicated here or referenced throughout the review are at different stages of clinical development for pancreatic cancer prevention and treatment. Ab, antibody; FR-α, folate receptor-α; TLR, Toll-like receptors.

As described above, the CCL2/CCR2 axis plays an important role in monocyte recruitment in many cancer types, including pancreatic cancer [144, 145]. Targeting the CCL2/CCR2 axis is promising as it results in blocking mobilization of monocytes from the bone marrow to the blood, which results in preventing their recruitment to the tumor [146] (Figure 3). In fact, CCL2 has been implicated in various neoplasias and adopted as a therapeutic target [40]. One of the CCL2-targeting agents, trabectedin, has been effectively used in clinic to treat human ovarian cancer and myxoid liposarcoma, as second-line treatment. In addition, antibodies (carlumab) to CCL2 are now being tested in clinical trials [147]. Combinations of carlumab with conventional chemotherapy regimens showed preliminary antitumor activity in advanced cancer patients and was well tolerated [148].

Additionally, PF-04136309, a small molecule CCR2 inhibitor, depletes CCR-2^+^/CD14^+^ monocytes and macrophages from the primary pancreatic tumor and premetastatic liver resulting in enhanced anti-tumor immunity, decreased tumor growth, and reduced metastasis in mice model [39]. Recently, we observed that the combination of metformin and aspirin dramatically down-regulated the mRNA expression of the CCL2 in treated pancreatic cancer cells (50-fold down-regulation vs. untreated cells), indicating a possibility of the use of metformin and aspirin as adjuvant cancer immunotherapy [149].

### Targeting macrophage activation

Substantial evidence suggests that inhibition of CSF-1/CSF-1R signaling by CSF1/CSF-1R neutralizing antibodies or CSF-1R1/CSF1R2 inhibitors (e.g. PLX6134/PLX3397) is one of the most advanced approaches to target macrophage activation [151, 152] (Figure 3). Lin *et al*. showed that CSF-1 can promote metastatic potential of breast cancer by regulating the infiltration and function of TAM, and inhibition of CSF-1 signaling in PyMT models could inhibit tumor progression and metastasis [153]. Similarly, DeNardo et al. showed that blockade of macrophage recruitment with CSF-1R signaling antagonists, in combination with paclitaxel, improved survival of mammary tumor-bearing mice by slowing primary tumor development and reducing pulmonary metastasis [154]. In glioblastoma mouse models, striking inhibition of CSF-1R could result in a dramatic reduction in tumor volume and long-term survival of the mice [155]. Interestingly, this CSF-1R inhibition did not kill the TAM but caused them to repolarize to a state regulated by CSF-1 that has been suggested to be antitumoral [156]. More recently, a monoclonal antibody (RG7155) directed against human diffuse-type giant cell tumors CSF-1R has been described which improves the objective clinical responses and increases the CD8^+^/CD4^+^ T cell ratio through potently and selectively inhibiting CSF-1R signaling by blocking receptor dimerization induced by CSF-1 [151]. In addition, BLZ945, a selective *c-Fms* kinase/CSF-1R inhibitor, has produced potent antitumor activities in various types of malignancies [157]. Pyonteck *et al*. [155] found that the BLZ945-primed macrophages create an immunogenic antitumor milieu as manifested by increased activation of tumor-specific CD8^+^ T cells. In addition, receptor tyrosine kinase inhibitors, such as imatinib, dasatinib, and sunitinib, are proposed to regulate the tumorigenic and immunosuppressive functions of TAM [158-160]. The data suggested that targeting the activation of macrophages emerged as an important part of combinatorial therapies in human cancers. Several clinical trials combining the use of CSF-1R inhibitors and chemotherapy for the treatment of metastatic cancers have been initiated [161-163].

In human pancreatic tumors, GM-CSF (or CSF-2), as described above, is prominently expressed, compared to CSF-1. However, limited studies have reported on the inhibition of CSF-2/CSF-2R signaling in pancreatic cancer. In contrast, targeting TAM by inhibiting CSF-1R has been reported to decrease the numbers of pancreatic tumor-initiating cells and improves chemotherapeutic efficacy *in vivo* [124]. Zhu *et al*. [164] also showed that blockade of CSF-1/CSF-1R signaling in pancreatic tumors depletes CD206^High^ TAM and reprograms remaining macrophages to support antitumor immunity. These data suggested that CSF-1/CSF-1R signaling may be an effective therapeutic target to reprogram the immunosuppressive microenvironment of human PDA tumors and more studies targeting CSF-2/CSF-2R signaling are warranted to enhance the efficacy of pancreatic cancer immunotherapy.

### Increasing antitumor macrophages by reprogramming macrophage polarization

Reprogramming tumor infiltrating myeloid cells towards an antitumor phenotype may have the potential to enhance T cell-mediated antitumor responses and improve the efficacy of immunotherapies [165, 166] (Figure 3). Toll-like receptors (TLR), which are primarily expressed on macrophages, has been most closely connected to inflammation-mediated carcinogenesis and tumor progression [104]. Application of TLR4 siRNA and neutralizing antibodies against TLR4 and IL-10 expressed on M2-polarized TAM markedly inhibited E-cadherin reduction and the upregulation of Snail and vimentin in pancreatic cancer cells [116]. PolyI:C, a dsRNA analog that binding TLR-3, can reverse protumor macrophages to antitumor phenotype [167]. CpG-oligodeoxynucleotide (ODN), a TLR9 ligand, combined with anti-IL-10R antibodies can promptly switch infiltrating macrophages infiltrate from M2 to M1 and trigger innate response debulking large tumors within 16 hours [168].

Maintaining the immunosuppressive phenotype of TAM is a new role for NF-κB signaling. When NF-κB signaling is inhibited, TAM become cytotoxic to tumor cells and switch to a “classically” activated phenotype [166]. The NF-κB pathway can be activated by using TLR agonists and anti-CD40 mAbs [169]. CD40, another macrophage cell surface marker, can inhibit cytotoxic functions. Beatty *et al*. demonstrated that, in a mouse model of PDA, CD40 agonist antibodies promote a remarkable antitumor effect and induced high expression of M1 markers (MHC class II and CD86) in macrophages [170]. Furthermore, CD40 agonist antibodies (CP-870,893) enhanced the efficacy of gemcitabine in a small cohort of patients with surgically incurable pancreatic cancer [171].

As mentioned above, HRG, a host-produced antiangiogenic and immunomodulatory factor [172], has been reported to promote anti-tumor immune responses and vessel normalization by skewing TAM polarization away from the M2- to a tumor-inhibiting M1-phenotype [63]. HRG binds thrombospondins (TSP), heparin, Fcγ receptors (FcγR) and other molecules, implicated in tumorigenesis. Furthermore, FcγR, expressed in macrophages, is found to be the potential player in skewing macrophages to an M2 phenotype [173], however, FcγR can also mediate activation of macrophage cytotoxicity induced by Trastuzumab [174]. These data indicated that HRG and its FcγR may open new avenues for more effective cancer treatment (Figure 3).

In addition, Kratochvill *et al*. [175] recently showed that TNF, which can be released by both M1 and M2 macrophages, is essential for suppressing the number of M2 tumor macrophages and blocks IL-13 production, a pro-M2 cytokine, from activated eosinophils. Peroxisome proliferator-activated receptor (PPAR)-γ agonists (thiazolidinediones) which have been used in the treatment of diabetes, were found to be linked to M2 polarization [176, 177] (Figure 3). Other therapeutic strategies that have been reported to affect macrophage polarization include zoledronicacid [178], statins [179], trabectedin [180], which shed new light on their mode of action. Taken together, reprogramming and reshaping deranged macrophage polarization is the holy grail of macrophage therapeutic targeting.

### Decreasing survival of TAM

Unlike switching the TAM phenotype so as to combat cancer, targeting the destruction of TAM is another promising strategy against cancer. Macrophage surface markers are very important as they can act as useful targets. Interestingly, folate receptor-α (FR-α) originally detected in placenta, spleen, bone marrow, and thymus [181] has recently been shown to be over-expressed on TAM, indicating that it might be a good target for decreasing survival of TAM. Within myeloid lineage, FR-α is expressed in neutrophils and can mediate folate binding in ovarian cancer-associated murine macrophages [182]. Specially, FR-α was only reported on activated, but not resting or quiescent myeloid cells (primarily monocytes and macrophages) [183]. Further, FR-α constitutes a marker for M2-polarized phenotype that promotes tumor growth and metastasis and correlates with a poor prognosis [9, 184]. In an experimental glioma mouse model, injection of the immunotoxin targeting FR-α expressing TAM significantly depleted TAM and reduced tumor growth [185] (Figure 3). Shen *et al*. recently reported that FR-α expression was more pronounced in cells within the stroma, primarily macrophages and macrophage like cells than cancer cells in 20 different human cancer types [186]. These data suggested that FR-α may constitute a good target for specific delivery of therapeutic agents to activated macrophages, and blocking FR-α expression by immunotoxin or other therapeutic agents may effectively decrease the survival of TAM, and thus reduce the tumor growth.

Interestingly, *Mycobacterium bovis bacillus* Calmette-Gue&acute;rin, which is used for the treatment of superficial bladder cancer via intravesical instillation, reduces tumor recurrence by stimulating the cytotoxic activity of macrophages [187]. In addition, certain bacteria that harbor in macrophages, such as *Listeria monocytogenes, Chlamydia psittaci* and *Legionella pneumophila*, are also being considered for TAM-targeted immunotherapy [188]. Intriguingly, certain sets of cytotoxic agents used in routine clinical practice, such as 5-FU and docetaxel, are found to have unique properties of selectively depleting M2 macrophages [189, 190] (Figure 3), while other cytotoxic agents, such as cisplatin, and paclitaxel, are proposed to trigger recruitment of macrophages into tumors by inducing CSF-1 and IL-34 expression [154]. PLX3397 (Plexxikon), which functions as a multi-kinase inhibitor targeting CSF-1R-associated kinases and c-kit, could also reduce survival of tumor-infiltrating M2 macrophages and increase infiltration of antitumor CTL in a syngeneic orthotopic murine model of mammary carcinogenesis [191]. Studies on macrophage distraction in pancreatic cancer are still in infancy, and further studies on this important and undeveloped research field are needed.

## CONCLUSIONS AND PERSPECTIVES

Pancreatic cancer is more aggressive than any other gastrointestinal malignancy due to the propensity for hematogenous or lymphatic metastasis in its early stage, which is associated with a poor prognosis. TAM, one of the major components of the tumor microenvironment, plays a critical role in pancreatic tumor progression. Tumors are heterogeneous, and numerous players shape TAM phenotypes and function, such as PPAR, STAT, NF-κB, and HIF families. Immunoprevention and immunotherapy by developing effective chemoprevention and therapeutic agents that can achieve an optimal balance between pro- and anti-tumor macrophage activities provides an opportunity for pancreatic cancer prevention and treatment. Among these strategies, re-educating TAM to have anti-tumorigenic effects, rather than targeting ablation *per se*, becomes a promising avenue of an effective therapeutic intervention. However, as indicated and referenced above, most strategies and agents to target the TAM in cancer have been evaluated only *in vivo* in mouse models, and anti-TAM therapy in the clinic is not well-established. Moreover, many of the emerging agents that have demonstrated efficacy in combating other types of tumors via modulation of macrophages in tumor microenvironments have not been systematically evaluated for pancreatic cancer prevention and treatment. Further investigation of these novel agents or monoclonal antibodies targeting TAM in human pancreatic cancer is warranted to with the hope of improving the outcome of this fatal disease.

## References

[1] Hidalgo M (2010). Pancreatic cancer. The New England journal of medicine.

[2] Cartwright T, Richards DA, Boehm KA (2008). Cancer of the pancreas: are we making progress? A review of studies in the US Oncology Research Network. Cancer control.

[3] Matrisian LM, Aizenberg R, A R (2012). The Alarming Rise of Pancreatic Cancer Deaths in the United States: Why We Need to Stem the Tide Today. Newsletter of the Pancreatic Cancer Action Network.

[4] Shakya R, Gonda T, Quante M, Salas M, Kim S, Brooks J, Hirsch S, Davies J, Cullo A, Olive K, Wang TC, Szabolcs M, Tycko B, Ludwig T (2013). Hypomethylating therapy in an aggressive stroma-rich model of pancreatic carcinoma. Cancer research.

[5] Mielgo A, Schmid MC (2013). Impact of tumour associated macrophages in pancreatic cancer. BMB reports.

[6] Sideras K, Braat H, Kwekkeboom J, van Eijck CH, Peppelenbosch MP, Sleijfer S, Bruno M (2014). Role of the immune system in pancreatic cancer progression and immune modulating treatment strategies. Cancer treatment reviews.

[7] Balkwill FR, Mantovani A (2012). Cancer-related inflammation: common themes and therapeutic opportunities. Seminars in cancer biology.

[8] Fan QM, Jing YY, Yu GF, Kou XR, Ye F, Gao L, Li R, Zhao QD, Yang Y, Lu ZH, Wei LX (2014). Tumor-associated macrophages promote cancer stem cell-like properties via transforming growth factor-beta1-induced epithelial-mesenchymal transition in hepatocellular carcinoma. Cancer letters.

[9] Kurahara H, Takao S, Kuwahata T, Nagai T, Ding Q, Maeda K, Shinchi H, Mataki Y, Maemura K, Matsuyama T, Natsugoe S (2012). Clinical significance of folate receptor beta-expressing tumor-associated macrophages in pancreatic cancer. Annals of surgical oncology.

[10] Segaliny AI, Mohamadi A, Dizier B, Lokajczyk A, Brion R, Lanel R, Amiaud J, Charrier C, Boisson-Vidal C, Heymann D (2014). Interleukin-34 promotes tumor progression and metastatic process in osteosarcoma through induction of angiogenesis and macrophage recruitment. International journal of cancer.

[11] Chen Q, Zhang XH, Massague J (2011). Macrophage binding to receptor VCAM-1 transmits survival signals in breast cancer cells that invade the lungs. Cancer cell.

[12] Qian BZ, Pollard JW (2010). Macrophage diversity enhances tumor progression and metastasis. Cell.

[13] Coussens LM, Zitvogel L, Palucka AK (2013). Neutralizing tumor-promoting chronic inflammation: a magic bullet?. Science.

[14] Farren MR, Mace TA, Geyer S, Mikhail S, Wu C, Ciombor K, Tahiri S, Ahn D, Noonan AM, Villalona-Calero M, Bekaii-Saab T, Lesinski GB (2015). Systemic Immune Activity Predicts Overall Survival in Treatment-Naive Patients with Metastatic Pancreatic Cancer. Clinical cancer research.

[15] Sierra JR, Corso S, Caione L, Cepero V, Conrotto P, Cignetti A, Piacibello W, Kumanogoh A, Kikutani H, Comoglio PM, Tamagnone L, Giordano S (2008). Tumor angiogenesis and progression are enhanced by Sema4D produced by tumor-associated macrophages. The Journal of experimental medicine.

[16] Fjallskog ML, Lejonklou MH, Oberg KE, Eriksson BK, Janson ET (2003). Expression of molecular targets for tyrosine kinase receptor antagonists in malignant endocrine pancreatic tumors. Clinical cancer research.

[17] Tugues S, Honjo S, Konig C, Noguer O, Hedlund M, Botling J, Deschoemaeker S, Wenes M, Rolny C, Jahnen-Dechent W, Mazzone M, Claesson-Welsh L (2012). Genetic deficiency in plasma protein HRG enhances tumor growth and metastasis by exacerbating immune escape and vessel abnormalization. Cancer research.

[18] Jinushi M, Chiba S, Yoshiyama H, Masutomi K, Kinoshita I, Dosaka-Akita H, Yagita H, Takaoka A, Tahara H (2011). Tumor-associated macrophages regulate tumorigenicity and anticancer drug responses of cancer stem/initiating cells. Proceedings of the National Academy of Sciences of the United States of America.

[19] Bingle L, Brown NJ, Lewis CE (2002). The role of tumour-associated macrophages in tumour progression: implications for new anticancer therapies. The Journal of pathology.

[20] Hu H, Hang JJ, Han T, Zhuo M, Jiao F, Wang LW (2016). The M2 phenotype of tumor-associated macrophages in the stroma confers a poor prognosis in pancreatic cancer. Tumour biology.

[21] Kurahara H, Shinchi H, Mataki Y, Maemura K, Noma H, Kubo F, Sakoda M, Ueno S, Natsugoe S, Takao S (2011). Significance of M2-polarized tumor-associated macrophage in pancreatic cancer. The Journal of surgical research.

[22] Meng F, Li C, Li W, Gao Z, Guo K, Song S (2014). Interaction between pancreatic cancer cells and tumor-associated macrophages promotes the invasion of pancreatic cancer cells and the differentiation and migration of macrophages. IUBMB life.

[23] Chen SJ, Zhang QB, Zeng LJ, Lian GD, Li JJ, Qian CC, Chen YZ, Chen YT, Huang KH (2015). Distribution and clinical significance of tumour-associated macrophages in pancreatic ductal adenocarcinoma: a retrospective analysis in China. Current oncology (Toronto, Ont).

[24] Sica A, Mantovani A (2012). Macrophage plasticity and polarization: in vivo veritas. The Journal of clinical investigation.

[25] Karnevi E, Andersson R, Rosendahl AH (2014). Tumour-educated macrophages display a mixed polarisation and enhance pancreatic cancer cell invasion. Immunology and cell biology.

[26] Helm O, Held-Feindt J, Grage-Griebenow E, Reiling N, Ungefroren H, Vogel I, Kruger U, Becker T, Ebsen M, Rocken C, Kabelitz D, Schafer H, Sebens S (2014). Tumor-associated macrophages exhibit pro- and anti-inflammatory properties by which they impact on pancreatic tumorigenesis. International journal of cancer.

[27] Lin EY, Li JF, Gnatovskiy L, Deng Y, Zhu L, Grzesik DA, Qian H, Xue XN, Pollard JW (2006). Macrophages regulate the angiogenic switch in a mouse model of breast cancer. Cancer research.

[28] Ruffell B, Affara NI, Coussens LM (2012). Differential macrophage programming in the tumor microenvironment. Trends in immunology.

[29] Klug F, Prakash H, Huber PE, Seibel T, Bender N, Halama N, Pfirschke C, Voss RH, Timke C, Umansky L, Klapproth K, Schakel K, Garbi N, Jager D, Weitz J, Schmitz-Winnenthal H (2013). Low-dose irradiation programs macrophage differentiation to an iNOS(+)/M1 phenotype that orchestrates effective T cell immunotherapy. Cancer cell.

[30] Di Caro G, Cortese N, Castino GF, Grizzi F, Gavazzi F, Ridolfi C, Capretti G, Mineri R, Todoric J, Zerbi A, Allavena P, Mantovani A, Marchesi F (2015). Dual prognostic significance of tumour-associated macrophages in human pancreatic adenocarcinoma treated or untreated with chemotherapy. Gut.

[31] Cortez-Retamozo V, Etzrodt M, Newton A, Rauch PJ, Chudnovskiy A, Berger C, Ryan RJ, Iwamoto Y, Marinelli B, Gorbatov R, Forghani R, Novobrantseva TI, Koteliansky V, Figueiredo JL, Chen JW, Anderson DG (2012). Origins of tumor-associated macrophages and neutrophils. Proceedings of the National Academy of Sciences of the United States of America.

[32] Franklin RA, Liao W, Sarkar A, Kim MV, Bivona MR, Liu K, Pamer EG, Li MO (2014). The cellular and molecular origin of tumor-associated macrophages. Science.

[33] Li S, Wang W, Zhang N, Ma T, Zhao C (2014). IL-1beta mediates MCP-1 induction by Wnt5a in gastric cancer cells. BMC cancer.

[34] Schumacher MA, Donnelly JM, Engevik AC, Xiao C, Yang L, Kenny S, Varro A, Hollande F, Samuelson LC, Zavros Y (2012). Gastric Sonic Hedgehog acts as a macrophage chemoattractant during the immune response to Helicobacter pylori. Gastroenterology.

[35] Oshima H, Hioki K, Popivanova BK, Oguma K, Van Rooijen N, Ishikawa TO, Oshima M (2011). Prostaglandin E(2) signaling and bacterial infection recruit tumor-promoting macrophages to mouse gastric tumors. Gastroenterology.

[36] Shi C, Pamer EG (2011). Monocyte recruitment during infection and inflammation. Nature reviews Immunology.

[37] Nakashima E, Mukaida N, Kubota Y, Kuno K, Yasumoto K, Ichimura F, Nakanishi I, Miyasaka M, Matsushima K (1995). Human MCAF gene transfer enhances the metastatic capacity of a mouse cachectic adenocarcinoma cell line in vivo. Pharmaceutical research.

[38] Huang S, Singh RK, Xie K, Gutman M, Berry KK, Bucana CD, Fidler IJ, Bar-Eli M (1994). Expression of the JE/MCP-1 gene suppresses metastatic potential in murine colon carcinoma cells. Cancer immunology, immunotherapy.

[39] Sanford DE, Belt BA, Panni RZ, Mayer A, Deshpande AD, Carpenter D, Mitchem JB, Plambeck-Suess SM, Worley LA, Goetz BD, Wang-Gillam A, Eberlein TJ, Denardo DG, Goedegebuure SP, Linehan DC (2013). Inflammatory monocyte mobilization decreases patient survival in pancreatic cancer: a role for targeting the CCL2/CCR2 axis. Clinical cancer research.

[40] Qian BZ, Li J, Zhang H, Kitamura T, Zhang J, Campion LR, Kaiser EA, Snyder LA, Pollard JW (2011). CCL2 recruits inflammatory monocytes to facilitate breast-tumour metastasis. Nature.

[41] Blanchard JA, Barve S, Joshi-Barve S, Talwalker R, Gates LK (2000). Cytokine production by CAPAN-1 and CAPAN-2 cell lines. Digestive diseases and sciences.

[42] Arlt A, Vorndamm J, Muerkoster S, Yu H, Schmidt WE, Folsch UR, Schafer H (2002). Autocrine production of interleukin 1beta confers constitutive nuclear factor kappaB activity and chemoresistance in pancreatic carcinoma cell lines. Cancer research.

[43] Negus RP, Stamp GW, Hadley J, Balkwill FR (1997). Quantitative assessment of the leukocyte infiltrate in ovarian cancer and its relationship to the expression of C-C chemokines. The American journal of pathology.

[44] Monti P, Leone BE, Marchesi F, Balzano G, Zerbi A, Scaltrini F, Pasquali C, Calori G, Pessi F, Sperti C, Di Carlo V, Allavena P, Piemonti L (2003). The CC chemokine MCP-1/CCL2 in pancreatic cancer progression: regulation of expression and potential mechanisms of antimalignant activity. Cancer research.

[45] Azenshtein E, Luboshits G, Shina S, Neumark E, Shahbazian D, Weil M, Wigler N, Keydar I, Ben-Baruch A (2002). The CC chemokine RANTES in breast carcinoma progression: regulation of expression and potential mechanisms of promalignant activity. Cancer research.

[46] Schlecker E, Stojanovic A, Eisen C, Quack C, Falk CS, Umansky V, Cerwenka A (1950). Tumor-infiltrating monocytic myeloid-derived suppressor cells mediate CCR5-dependent recruitment of regulatory T cells favoring tumor growth. Journal of immunology.

[47] Van Overmeire E, Laoui D, Keirsse J, Van Ginderachter JA, Sarukhan A (2014). Mechanisms driving macrophage diversity and specialization in distinct tumor microenvironments and parallelisms with other tissues. Frontiers in immunology.

[48] Locati M, Deuschle U, Massardi ML, Martinez FO, Sironi M, Sozzani S, Bartfai T, Mantovani A (1950). Analysis of the gene expression profile activated by the CC chemokine ligand 5/RANTES and by lipopolysaccharide in human monocytes. Journal of immunology.

[49] Curiel TJ, Coukos G, Zou L, Alvarez X, Cheng P, Mottram P, Evdemon-Hogan M, Conejo-Garcia JR, Zhang L, Burow M, Zhu Y, Wei S, Kryczek I, Daniel B, Gordon A, Myers L (2004). Specific recruitment of regulatory T cells in ovarian carcinoma fosters immune privilege and predicts reduced survival. Nature medicine.

[50] Adeegbe DO, Nishikawa H (2013). Natural and induced T regulatory cells in cancer. Frontiers in immunology.

[51] Biswas SK, Gangi L, Paul S, Schioppa T, Saccani A, Sironi M, Bottazzi B, Doni A, Vincenzo B, Pasqualini F, Vago L, Nebuloni M, Mantovani A, Sica A (2006). A distinct and unique transcriptional program expressed by tumor-associated macrophages (defective NF-kappaB and enhanced IRF-3/STAT1 activation). Blood.

[52] Liu J, Zhang N, Li Q, Zhang W, Ke F, Leng Q, Wang H, Chen J, Wang H (2011). Tumor-associated macrophages recruit CCR6+ regulatory T cells and promote the development of colorectal cancer via enhancing CCL20 production in mice. PloS one.

[53] Lin EY, Li JF, Bricard G, Wang W, Deng Y, Sellers R, Porcelli SA, Pollard JW (2007). Vascular endothelial growth factor restores delayed tumor progression in tumors depleted of macrophages. Molecular oncology.

[54] Panni RZ, Linehan DC, DeNardo DG (2013). Targeting tumor-infiltrating macrophages to combat cancer. Immunotherapy.

[55] Bayne LJ, Beatty GL, Jhala N, Clark CE, Rhim AD, Stanger BZ, Vonderheide RH (2012). Tumor-derived granulocyte-macrophage colony-stimulating factor regulates myeloid inflammation and T cell immunity in pancreatic cancer. Cancer cell.

[56] Linde N, Lederle W, Depner S, van Rooijen N, Gutschalk CM, Mueller MM (2012). Vascular endothelial growth factor-induced skin carcinogenesis depends on recruitment and alternative activation of macrophages. The Journal of pathology.

[57] Uutela M, Wirzenius M, Paavonen K, Rajantie I, He Y, Karpanen T, Lohela M, Wiig H, Salven P, Pajusola K, Eriksson U, Alitalo K (2004). PDGF-D induces macrophage recruitment, increased interstitial pressure, and blood vessel maturation during angiogenesis. Blood.

[58] Olson P, Chu GC, Perry SR, Nolan-Stevaux O, Hanahan D (2011). Imaging guided trials of the angiogenesis inhibitor sunitinib in mouse models predict efficacy in pancreatic neuroendocrine but not ductal carcinoma. Proceedings of the National Academy of Sciences of the United States of America.

[59] Lawrence T, Natoli G (2011). Transcriptional regulation of macrophage polarization: enabling diversity with identity. Nature reviews Immunology.

[60] Martinez FO, Helming L, Gordon S (2009). Alternative activation of macrophages: an immunologic functional perspective. Annual review of immunology.

[61] Van Overmeire E, Stijlemans B, Heymann F, Keirsse J, Morias Y, Elkrim Y, Brys L, Abels C, Lahmar Q, Ergen C, Vereecke L, Tacke F, De Baetselier P, Van Ginderachter JA, Laoui D (2015). M-CSF and GM-CSF receptor signaling differentially regulate monocyte maturation and macrophage polarization in the tumor microenvironment. Cancer research.

[62] Takeda N, O'Dea EL, Doedens A, Kim JW, Weidemann A, Stockmann C, Asagiri M, Simon MC, Hoffmann A, Johnson RS (2010). Differential activation and antagonistic function of HIF-{alpha} isoforms in macrophages are essential for NO homeostasis. Genes & development.

[63] Rolny C, Mazzone M, Tugues S, Laoui D, Johansson I, Coulon C, Squadrito ML, Segura I, Li X, Knevels E, Costa S, Vinckier S, Dresselaer T, Akerud P, De Mol M, Salomaki H (2011). HRG inhibits tumor growth and metastasis by inducing macrophage polarization and vessel normalization through downregulation of PlGF. Cancer cell.

[64] Hermano E, Meirovitz A, Meir K, Nussbaum G, Appelbaum L, Peretz T, Elkin M (2014). Macrophage polarization in pancreatic carcinoma: role of heparanase enzyme. Journal of the National Cancer Institute.

[65] Gironella M, Calvo C, Fernandez A, Closa D, Iovanna JL, Rosello-Catafau J, Folch-Puy E (2013). Reg3beta deficiency impairs pancreatic tumor growth by skewing macrophage polarization. Cancer research.

[66] Kuhnemuth B, Muhlberg L, Schipper M, Griesmann H, Neesse A, Milosevic N, Wissniowski T, Buchholz M, Gress TM, Michl P (2015). CUX1 modulates polarization of tumor-associated macrophages by antagonizing NF-kappaB signaling. Oncogene.

[67] Riabov V, Gudima A, Wang N, Mickley A, Orekhov A, Kzhyshkowska J (2014). Role of tumor associated macrophages in tumor angiogenesis and lymphangiogenesis. Frontiers in physiology.

[68] Whipple C, Korc M (2008). Targeting angiogenesis in pancreatic cancer: rationale and pitfalls. Langenbeck's archives of surgery.

[69] Dineen SP, Lynn KD, Holloway SE, Miller AF, Sullivan JP, Shames DS, Beck AW, Barnett CC, Fleming JB, Brekken RA (2008). Vascular endothelial growth factor receptor 2 mediates macrophage infiltration into orthotopic pancreatic tumors in mice. Cancer research.

[70] Xie K, Wei D, Huang S (2006). Transcriptional anti-angiogenesis therapy of human pancreatic cancer. Cytokine & growth factor reviews.

[71] Thompson WD, Smith EB, Stirk CM, Marshall FI, Stout AJ, Kocchar A (1992). Angiogenic activity of fibrin degradation products is located in fibrin fragment E. The Journal of pathology.

[72] Wang FQ, So J, Reierstad S, Fishman DA (2005). Matrilysin (MMP-7) promotes invasion of ovarian cancer cells by activation of progelatinase. International journal of cancer.

[73] Pepper MS (2001). Role of the matrix metalloproteinase and plasminogen activator-plasmin systems in angiogenesis. Arteriosclerosis, thrombosis, and vascular biology.

[74] Lakka SS, Gondi CS, Dinh DH, Olivero WC, Gujrati M, Rao VH, Sioka C, Rao JS (2005). Specific interference of urokinase-type plasminogen activator receptor and matrix metalloproteinase-9 gene expression induced by double-stranded RNA results in decreased invasion, tumor growth, and angiogenesis in gliomas. The Journal of biological chemistry.

[75] Gondi CS, Lakka SS, Dinh DH, Olivero WC, Gujrati M, Rao JS (2007). Intraperitoneal injection of a hairpin RNA-expressing plasmid targeting urokinase-type plasminogen activator (uPA) receptor and uPA retards angiogenesis and inhibits intracranial tumor growth in nude mice. Clinical cancer research.

[76] Kaneko T, Konno H, Baba M, Tanaka T, Nakamura S (2003). Urokinase-type plasminogen activator expression correlates with tumor angiogenesis and poor outcome in gastric cancer. Cancer science.

[77] Feig C, Gopinathan A, Neesse A, Chan DS, Cook N, Tuveson DA (2012). The pancreas cancer microenvironment. Clinical cancer research.

[78] Olive KP, Jacobetz MA, Davidson CJ, Gopinathan A, McIntyre D, Honess D, Madhu B, Goldgraben MA, Caldwell ME, Allard D, Frese KK, Denicola G, Feig C, Combs C, Winter SP, Ireland-Zecchini H (2009). Inhibition of Hedgehog signaling enhances delivery of chemotherapy in a mouse model of pancreatic cancer. Science.

[79] Michalski CW, Erkan M, Friess H, Kleeff J (2010). Tumor metabolism to blood flow ratio in pancreatic cancer: helpful in patient stratification?. Future oncology (London, England).

[80] Nguyen NC, Taalab K, Osman MM (2010). Decreased blood flow with increased metabolic activity: a novel sign of pancreatic tumor aggressiveness. Clinical cancer research.

[81] Vaupel P, Mayer A (2007). Hypoxia in cancer: significance and impact on clinical outcome. Cancer metastasis reviews.

[82] Kumar V, Gabrilovich DI (2014). Hypoxia-inducible factors in regulation of immune responses in tumour microenvironment. Immunology.

[83] Werno C, Menrad H, Weigert A, Dehne N, Goerdt S, Schledzewski K, Kzhyshkowska J, Brune B (2010). Knockout of HIF-1alpha in tumor-associated macrophages enhances M2 polarization and attenuates their pro-angiogenic responses. Carcinogenesis.

[84] Zhang L, Zhou F, Garcia de Vinuesa A, de Kruijf EM, Mesker WE, Hui L, Drabsch Y, Li Y, Bauer A, Rousseau A, Sheppard KA, Mickanin C, Kuppen PJ, Lu CX, Ten Dijke P (2013). TRAF4 promotes TGF-beta receptor signaling and drives breast cancer metastasis. Molecular cell.

[85] Oshimori N, Oristian D, Fuchs E (2015). TGF-beta Promotes Heterogeneity and Drug Resistance in Squamous Cell Carcinoma. Cell.

[86] Jeon SH, Chae BC, Kim HA, Seo GY, Seo DW, Chun GT, Kim NS, Yie SW, Byeon WH, Eom SH, Ha KS, Kim YM, Kim PH (2007). Mechanisms underlying TGF-beta1-induced expression of VEGF and Flk-1 in mouse macrophages and their implications for angiogenesis. Journal of leukocyte biology.

[87] Shen Z, Seppanen H, Kauttu T, Vainionpaa S, Ye Y, Wang S, Mustonen H, Puolakkainen P (2013). Vasohibin-1 expression is regulated by transforming growth factor-beta/bone morphogenic protein signaling pathway between tumor-associated macrophages and pancreatic cancer cells. Journal of interferon & cytokine research.

[88] Mazzieri R, Pucci F, Moi D, Zonari E, Ranghetti A, Berti A, Politi LS, Gentner B, Brown JL, Naldini L, De Palma M (2011). Targeting the ANG2/TIE2 axis inhibits tumor growth and metastasis by impairing angiogenesis and disabling rebounds of proangiogenic myeloid cells. Cancer cell.

[89] Daly C, Eichten A, Castanaro C, Pasnikowski E, Adler A, Lalani AS, Papadopoulos N, Kyle AH, Minchinton AI, Yancopoulos GD, Thurston G (2013). Angiopoietin-2 functions as a Tie2 agonist in tumor models, where it limits the effects of VEGF inhibition. Cancer research.

[90] Venneri MA, De Palma M, Ponzoni M, Pucci F, Scielzo C, Zonari E, Mazzieri R, Doglioni C, Naldini L (2007). Identification of proangiogenic TIE2-expressing monocytes (TEMs) in human peripheral blood and cancer. Blood.

[91] De Palma M, Venneri MA, Galli R, Sergi Sergi L, Politi LS, Sampaolesi M, Naldini L (2005). Tie2 identifies a hematopoietic lineage of proangiogenic monocytes required for tumor vessel formation and a mesenchymal population of pericyte progenitors. Cancer cell.

[92] De Palma M, Venneri MA, Roca C, Naldini L (2003). Targeting exogenous genes to tumor angiogenesis by transplantation of genetically modified hematopoietic stem cells. Nature medicine.

[93] Lewis CE, De Palma M, Naldini L (2007). Tie2-expressing monocytes and tumor angiogenesis: regulation by hypoxia and angiopoietin-2. Cancer research.

[94] Forget MA, Voorhees JL, Cole SL, Dakhlallah D, Patterson IL, Gross AC, Moldovan L, Mo X, Evans R, Marsh CB, Eubank TD (2014). Macrophage colony-stimulating factor augments Tie2-expressing monocyte differentiation, angiogenic function, and recruitment in a mouse model of breast cancer. PloS one.

[95] Ji RC (2006). Lymphatic endothelial cells, tumor lymphangiogenesis and metastasis: New insights into intratumoral and peritumoral lymphatics. Cancer metastasis reviews.

[96] Schoppmann SF, Birner P, Stockl J, Kalt R, Ullrich R, Caucig C, Kriehuber E, Nagy K, Alitalo K, Kerjaschki D (2002). Tumor-associated macrophages express lymphatic endothelial growth factors and are related to peritumoral lymphangiogenesis. The American journal of pathology.

[97] Cursiefen C, Chen L, Borges LP, Jackson D, Cao J, Radziejewski C, D'Amore PA, Dana MR, Wiegand SJ, Streilein JW (2004). VEGF-A stimulates lymphangiogenesis and hemangiogenesis in inflammatory neovascularization via macrophage recruitment. The Journal of clinical investigation.

[98] Hao NB, Lu MH, Fan YH, Cao YL, Zhang ZR, Yang SM (2012). Macrophages in tumor microenvironments and the progression of tumors. Clinical & developmental immunology.

[99] Doedens AL, Stockmann C, Rubinstein MP, Liao D, Zhang N, DeNardo DG, Coussens LM, Karin M, Goldrath AW, Johnson RS (2010). Macrophage expression of hypoxia-inducible factor-1 alpha suppresses T-cell function and promotes tumor progression. Cancer research.

[100] Laoui D, Van Overmeire E, Di Conza G, Aldeni C, Keirsse J, Morias Y, Movahedi K, Houbracken I, Schouppe E, Elkrim Y, Karroum O, Jordan B, Carmeliet P, Gysemans C, De Baetselier P, Mazzone M (2014). Tumor hypoxia does not drive differentiation of tumor-associated macrophages but rather fine-tunes the M2-like macrophage population. Cancer research.

[101] Mazzone M, Dettori D, Leite de Oliveira R, Loges S, Schmidt T, Jonckx B, Tian YM, Lanahan AA, Pollard P, Ruiz de Almodovar C, De Smet F, Vinckier S, Aragones J, Debackere K, Luttun A, Wyns S (2009). Heterozygous deficiency of PHD2 restores tumor oxygenation and inhibits metastasis via endothelial normalization. Cell.

[102] Van Overmeire E, Laoui D, Keirsse J, Van Ginderachter JA (2014). Hypoxia and tumor-associated macrophages: A deadly alliance in support of tumor progression. Oncoimmunology.

[103] Kuang DM, Zhao Q, Peng C, Xu J, Zhang JP, Wu C, Zheng L (2009). Activated monocytes in peritumoral stroma of hepatocellular carcinoma foster immune privilege and disease progression through PD-L1. The Journal of experimental medicine.

[104] Plate JM, Harris JE (2000). Immune cell functions in pancreatic cancer. Critical reviews in immunology.

[105] Yamamoto T, Yanagimoto H, Satoi S, Toyokawa H, Hirooka S, Yamaki S, Yui R, Yamao J, Kim S, Kwon AH (2012). Circulating CD4+CD25+ regulatory T cells in patients with pancreatic cancer. Pancreas.

[106] Liyanage UK, Moore TT, Joo HG, Tanaka Y, Herrmann V, Doherty G, Drebin JA, Strasberg SM, Eberlein TJ, Goedegebuure PS, Linehan DC (1950). Prevalence of regulatory T cells is increased in peripheral blood and tumor microenvironment of patients with pancreas or breast adenocarcinoma. Journal of immunology.

[107] Gabrilovich DI, Nagaraj S (2009). Myeloid-derived suppressor cells as regulators of the immune system. Nature reviews Immunology.

[108] Mantovani A, Schioppa T, Porta C, Allavena P, Sica A (2006). Role of tumor-associated macrophages in tumor progression and invasion. Cancer metastasis reviews.

[109] Bronte V, Zanovello P (2005). Regulation of immune responses by L-arginine metabolism. Nature reviews Immunology.

[110] Li MO, Flavell RA (2008). TGF-beta: a master of all T cell trades. Cell.

[111] Shevach EM, Davidson TS, Huter EN, Dipaolo RA, Andersson J (2008). Role of TGF-Beta in the induction of Foxp3 expression and T regulatory cell function. Journal of clinical immunology.

[112] Clark CE, Hingorani SR, Mick R, Combs C, Tuveson DA, Vonderheide RH (2007). Dynamics of the immune reaction to pancreatic cancer from inception to invasion. Cancer research.

[113] Soares KC, Rucki AA, Wu AA, Olino K, Xiao Q, Chai Y, Wamwea A, Bigelow E, Lutz E, Liu L, Yao S, Anders RA, Laheru D, Wolfgang CL, Edil BH, Schulick RD (1997). PD-1/PD-L1 blockade together with vaccine therapy facilitates effector T-cell infiltration into pancreatic tumors. Journal of immunotherapy.

[114] Biddle A, Mackenzie IC (2012). Cancer stem cells and EMT in carcinoma. Cancer metastasis reviews.

[115] Javle MM, Gibbs JF, Iwata KK, Pak Y, Rutledge P, Yu J, Black JD, Tan D, Khoury T (2007). Epithelial-mesenchymal transition (EMT) and activated extracellular signal-regulated kinase (p-Erk) in surgically resected pancreatic cancer. Annals of surgical oncology.

[116] Liu CY, Xu JY, Shi XY, Huang W, Ruan TY, Xie P, Ding JL (2013). M2-polarized tumor-associated macrophages promoted epithelial-mesenchymal transition in pancreatic cancer cells, partially through TLR4/IL-10 signaling pathway. Laboratory investigation; a journal of technical methods and pathology.

[117] Bonde AK, Tischler V, Kumar S, Soltermann A, Schwendener RA (2012). Intratumoral macrophages contribute to epithelial-mesenchymal transition in solid tumors. BMC cancer.

[118] Fu XT, Dai Z, Song K, Zhang ZJ, Zhou ZJ, Zhou SL, Zhao YM, Xiao YS, Sun QM, Ding ZB, Fan J (2015). Macrophage-secreted IL-8 induces epithelial-mesenchymal transition in hepatocellular carcinoma cells by activating the JAK2/STAT3/Snail pathway. International journal of oncology.

[119] Su S, Liu Q, Chen J, Chen J, Chen F, He C, Huang D, Wu W, Lin L, Huang W, Zhang J, Cui X, Zheng F, Li H, Yao H, Su F (2014). A positive feedback loop between mesenchymal-like cancer cells and macrophages is essential to breast cancer metastasis. Cancer cell.

[120] Funamizu N, Hu C, Lacy C, Schetter A, Zhang G, He P, Gaedcke J, Ghadimi MB, Ried T, Yfantis HG, Lee DH, Subleski J, Chan T, Weiss JM, Back TC, Yanaga K (2012). Macrophage migration inhibitory factor induces epithelial to mesenchymal transition, enhances tumor aggressiveness and predicts clinical outcome in resected pancreatic ductal adenocarcinoma. International journal of cancer.

[121] Meng F, Li W, Li C, Gao Z, Guo K, Song S (2015). CCL18 promotes epithelial-mesenchymal transition, invasion and migration of pancreatic cancer cells in pancreatic ductal adenocarcinoma. International journal of oncology.

[122] Hermann PC, Huber SL, Herrler T, Aicher A, Ellwart JW, Guba M, Bruns CJ, Heeschen C (2007). Distinct populations of cancer stem cells determine tumor growth and metastatic activity in human pancreatic cancer. Cell stem cell.

[123] Mueller MT, Hermann PC, Witthauer J, Rubio-Viqueira B, Leicht SF, Huber S, Ellwart JW, Mustafa M, Bartenstein P, D'Haese JG, Schoenberg MH, Berger F, Jauch KW, Hidalgo M, Heeschen C (2009). Combined targeted treatment to eliminate tumorigenic cancer stem cells in human pancreatic cancer. Gastroenterology.

[124] Mitchem JB, Brennan DJ, Knolhoff BL, Belt BA, Zhu Y, Sanford DE, Belaygorod L, Carpenter D, Collins L, Piwnica-Worms D, Hewitt S, Udupi GM, Gallagher WM, Wegner C, West BL, Wang-Gillam A (2013). Targeting tumor-infiltrating macrophages decreases tumor-initiating cells, relieves immunosuppression, and improves chemotherapeutic responses. Cancer research.

[125] Sainz B, Martin B, Tatari M, Heeschen C, Guerra S (2014). ISG15 is a critical microenvironmental factor for pancreatic cancer stem cells. Cancer research.

[126] Brown EJ, Frazier WA (2001). Integrin-associated protein (CD47) and its ligands. Trends in cell biology.

[127] Cioffi M, Trabulo SM, Hidalgo M, Costello E, Greenhalf W, Erkan M, Kleeff J, Sainz B, Heeschen C (2015). Inhibition of CD47 effectively targets pancreatic cancer stem cells via dual mechanism. Clinical cancer research.

[128] Sainz B, Alcala S, Garcia E, Sanchez-Ripoll Y, Azevedo MM, Cioffi M, Tatari M, Miranda-Lorenzo I, Hidalgo M, Gomez-Lopez G, Canamero M, Erkan M, Kleeff J, Garcia-Silva S, Sancho P, Hermann PC (2015). Microenvironmental hCAP-18/LL-37 promotes pancreatic ductal adenocarcinoma by activating its cancer stem cell compartment. Gut.

[129] Kleeff J, Kusama T, Rossi DL, Ishiwata T, Maruyama H, Friess H, Buchler MW, Zlotnik A, Korc M (1999). Detection and localization of Mip-3alpha/LARC/Exodus, a macrophage proinflammatory chemokine, and its CCR6 receptor in human pancreatic cancer. International journal of cancer.

[130] Campbell AS, Albo D, Kimsey TF, White SL, Wang TN (2005). Macrophage inflammatory protein-3alpha promotes pancreatic cancer cell invasion. The Journal of surgical research.

[131] Kimsey TF, Campbell AS, Albo D, Wilson M, Wang TN (2004). Co-localization of macrophage inflammatory protein-3alpha (Mip-3alpha) and its receptor, CCR6, promotes pancreatic cancer cell invasion. Cancer journal (Sudbury, Mass).

[132] Matsuo Y, Ochi N, Sawai H, Yasuda A, Takahashi H, Funahashi H, Takeyama H, Tong Z, Guha S (2009). CXCL8/IL-8 and CXCL12/SDF-1alpha co-operatively promote invasiveness and angiogenesis in pancreatic cancer. International journal of cancer.

[133] Schoppmann SF, Fenzl A, Nagy K, Unger S, Bayer G, Geleff S, Gnant M, Horvat R, Jakesz R, Birner P (2006). VEGF-C expressing tumor-associated macrophages in lymph node positive breast cancer: impact on lymphangiogenesis and survival. Surgery.

[134] Zumsteg A, Baeriswyl V, Imaizumi N, Schwendener R, Ruegg C, Christofori G (2009). Myeloid cells contribute to tumor lymphangiogenesis. PloS one.

[135] Kurahara H, Takao S, Maemura K, Mataki Y, Kuwahata T, Maeda K, Sakoda M, Iino S, Ishigami S, Ueno S, Shinchi H, Natsugoe S (2013). M2-polarized tumor-associated macrophage infiltration of regional lymph nodes is associated with nodal lymphangiogenesis and occult nodal involvement in pN0 pancreatic cancer. Pancreas.

[136] Cavel O, Shomron O, Shabtay A, Vital J, Trejo-Leider L, Weizman N, Krelin Y, Fong Y, Wong RJ, Amit M, Gil Z (2012). Endoneurial macrophages induce perineural invasion of pancreatic cancer cells by secretion of GDNF and activation of RET tyrosine kinase receptor. Cancer research.

[137] Takahashi M (2001). The GDNF/RET signaling pathway and human diseases. Cytokine & growth factor reviews.

[138] Veit C, Genze F, Menke A, Hoeffert S, Gress TM, Gierschik P, Giehl K (2004). Activation of phosphatidylinositol 3-kinase and extracellular signal-regulated kinase is required for glial cell line-derived neurotrophic factor-induced migration and invasion of pancreatic carcinoma cells. Cancer research.

[139] Mulligan LM (2014). RET revisited: expanding the oncogenic portfolio. Nature reviews Cancer.

[140] Ito Y, Okada Y, Sato M, Sawai H, Funahashi H, Murase T, Hayakawa T, Manabe T (2005). Expression of glial cell line-derived neurotrophic factor family members and their receptors in pancreatic cancers. Surgery.

[141] Zeng Q, Cheng Y, Zhu Q, Yu Z, Wu X, Huang K, Zhou M, Han S, Zhang Q (2008). The relationship between overexpression of glial cell-derived neurotrophic factor and its RET receptor with progression and prognosis of human pancreatic cancer. The Journal of international medical research.

[142] Gil Z, Cavel O, Kelly K, Brader P, Rein A, Gao SP, Carlson DL, Shah JP, Fong Y, Wong RJ (2010). Paracrine regulation of pancreatic cancer cell invasion by peripheral nerves. Journal of the National Cancer Institute.

[143] He S, Chen CH, Chernichenko N, He S, Bakst RL, Barajas F, Deborde S, Allen PJ, Vakiani E, Yu Z, Wong RJ (2014). GFRalpha1 released by nerves enhances cancer cell perineural invasion through GDNF-RET signaling. Proceedings of the National Academy of Sciences of the United States of America.

[144] Wolf MJ, Hoos A, Bauer J, Boettcher S, Knust M, Weber A, Simonavicius N, Schneider C, Lang M, Sturzl M, Croner RS, Konrad A, Manz MG, Moch H, Aguzzi A, van Loo G (2012). Endothelial CCR2 signaling induced by colon carcinoma cells enables extravasation via the JAK2-Stat5 and p38MAPK pathway. Cancer cell.

[145] Bonapace L, Coissieux MM, Wyckoff J, Mertz KD, Varga Z, Junt T, Bentires-Alj M (2014). Cessation of CCL2 inhibition accelerates breast cancer metastasis by promoting angiogenesis. Nature.

[146] Zhu X, Fujita M, Snyder LA, Okada H (2011). Systemic delivery of neutralizing antibody targeting CCL2 for glioma therapy. Journal of neuro-oncology.

[147] Sandhu SK, Papadopoulos K, Fong PC, Patnaik A, Messiou C, Olmos D, Wang G, Tromp BJ, Puchalski TA, Balkwill F, Berns B, Seetharam S, de Bono JS, Tolcher AW (2013). A first-in-human, first-in-class, phase I study of carlumab (CNTO 888), a human monoclonal antibody against CC-chemokine ligand 2 in patients with solid tumors. Cancer chemotherapy and pharmacology.

[148] Brana I, Calles A, LoRusso PM, Yee LK, Puchalski TA, Seetharam S, Zhong B, de Boer CJ, Tabernero J, Calvo E (2015). Carlumab, an anti-C-C chemokine ligand 2 monoclonal antibody, in combination with four chemotherapy regimens for the treatment of patients with solid tumors: an open-label, multicenter phase 1b study. Targeted oncology.

[149] Yue W, Wang T, Zachariah E, Lin Y, Yang CS, Xu Q, DiPaola RS, Tan XL (2015). Transcriptomic analysis of pancreatic cancer cells in response to metformin and aspirin: an implication of synergy. Scientific reports.

[150] Gabrilovich DI, Ostrand-Rosenberg S, Bronte V (2012). Coordinated regulation of myeloid cells by tumours. Nature reviews Immunology.

[151] Ries CH, Cannarile MA, Hoves S, Benz J, Wartha K, Runza V, Rey-Giraud F, Pradel LP, Feuerhake F, Klaman I, Jones T, Jucknischke U, Scheiblich S, Kaluza K, Gorr IH, Walz A (2014). Targeting tumor-associated macrophages with anti-CSF-1R antibody reveals a strategy for cancer therapy. Cancer cell.

[152] Mouchemore KA, Sampaio NG, Murrey MW, Stanley ER, Lannutti BJ, Pixley FJ (2013). Specific inhibition of PI3K p110delta inhibits CSF-1-induced macrophage spreading and invasive capacity. The FEBS journal.

[153] Lin EY, Nguyen AV, Russell RG, Pollard JW (2001). Colony-stimulating factor 1 promotes progression of mammary tumors to malignancy. The Journal of experimental medicine.

[154] DeNardo DG, Brennan DJ, Rexhepaj E, Ruffell B, Shiao SL, Madden SF, Gallagher WM, Wadhwani N, Keil SD, Junaid SA, Rugo HS, Hwang ES, Jirstrom K, West BL, Coussens LM (2011). Leukocyte complexity predicts breast cancer survival and functionally regulates response to chemotherapy. Cancer discovery.

[155] Pyonteck SM, Akkari L, Schuhmacher AJ, Bowman RL, Sevenich L, Quail DF, Olson OC, Quick ML, Huse JT, Teijeiro V, Setty M, Leslie CS, Oei Y, Pedraza A, Zhang J, Brennan CW (2013). CSF-1R inhibition alters macrophage polarization and blocks glioma progression. Nature medicine.

[156] Klemm F, Joyce JA (2015). Microenvironmental regulation of therapeutic response in cancer. Trends in cell biology.

[157] Strachan DC, Ruffell B, Oei Y, Bissell MJ, Coussens LM, Pryer N, Daniel D (2013). CSF1R inhibition delays cervical and mammary tumor growth in murine models by attenuating the turnover of tumor-associated macrophages and enhancing infiltration by CD8 T cells. Oncoimmunology.

[158] Dewar AL, Cambareri AC, Zannettino AC, Miller BL, Doherty KV, Hughes TP, Lyons AB (2005). Macrophage colony-stimulating factor receptor c-fms is a novel target of imatinib. Blood.

[159] Brownlow N, Mol C, Hayford C, Ghaem-Maghami S, Dibb NJ (2009). Dasatinib is a potent inhibitor of tumour-associated macrophages, osteoclasts and the FMS receptor. Leukemia.

[160] Faivre S, Demetri G, Sargent W, Raymond E (2007). Molecular basis for sunitinib efficacy and future clinical development. Nature reviews Drug discovery.

[161] West RB, Rubin BP, Miller MA, Subramanian S, Kaygusuz G, Montgomery K, Zhu S, Marinelli RJ, De Luca A, Downs-Kelly E, Goldblum JR, Corless CL, Brown PO, Gilks CB, Nielsen TO, Huntsman D (2006). A landscape effect in tenosynovial giant-cell tumor from activation of CSF1 expression by a translocation in a minority of tumor cells. Proceedings of the National Academy of Sciences of the United States of America.

[162] A Study of IMC-CS4 in Subjects With Advanced Solid Tumors http://www.clinicaltrials.gov/show/NCT01346358.

[163] Safety Study of PLX108-01 in Patients With Solid Tumors https://www.clinicaltrials.gov/show/NCT01004861.

[164] Zhu Y, Knolhoff BL, Meyer MA, Nywening TM, West BL, Luo J, Wang-Gillam A, Goedegebuure SP, Linehan DC, DeNardo DG (2014). CSF1/CSF1R blockade reprograms tumor-infiltrating macrophages and improves response to T-cell checkpoint immunotherapy in pancreatic cancer models. Cancer research.

[165] Jaiswal S, Chao MP, Majeti R, Weissman IL (2010). Macrophages as mediators of tumor immunosurveillance. Trends in immunology.

[166] Hagemann T, Lawrence T, McNeish I, Charles KA, Kulbe H, Thompson RG, Robinson SC, Balkwill FR (2008). “Re-educating” tumor-associated macrophages by targeting NF-kappaB. The Journal of experimental medicine.

[167] Shime H, Matsumoto M, Oshiumi H, Tanaka S, Nakane A, Iwakura Y, Tahara H, Inoue N, Seya T (2012). Toll-like receptor 3 signaling converts tumor-supporting myeloid cells to tumoricidal effectors. Proceedings of the National Academy of Sciences of the United States of America.

[168] Guiducci C, Vicari AP, Sangaletti S, Trinchieri G, Colombo MP (2005). Redirecting in vivo elicited tumor infiltrating macrophages and dendritic cells towards tumor rejection. Cancer research.

[169] Seya T, Shime H, Matsumoto M (2012). TAMable tumor-associated macrophages in response to innate RNA sensing. Oncoimmunology.

[170] Beatty GL, Chiorean EG, Fishman MP, Saboury B, Teitelbaum UR, Sun W, Huhn RD, Song W, Li D, Sharp LL, Torigian DA, O'Dwyer PJ, Vonderheide RH (2011). CD40 agonists alter tumor stroma and show efficacy against pancreatic carcinoma in mice and humans. Science.

[171] Beatty GL, Torigian DA, Chiorean EG, Saboury B, Brothers A, Alavi A, Troxel AB, Sun W, Teitelbaum UR, Vonderheide RH, O'Dwyer PJ (2013). A phase I study of an agonist CD40 monoclonal antibody (CP-870,893) in combination with gemcitabine in patients with advanced pancreatic ductal adenocarcinoma. Clinical cancer research.

[172] Blank M, Shoenfeld Y (2008). Histidine-rich glycoprotein modulation of immune/autoimmune, vascular, and coagulation systems. Clinical reviews in allergy & immunology.

[173] Andreu P, Johansson M, Affara NI, Pucci F, Tan T, Junankar S, Korets L, Lam J, Tawfik D, DeNardo DG, Naldini L, de Visser KE, De Palma M, Coussens LM (2010). FcRgamma activation regulates inflammation-associated squamous carcinogenesis. Cancer cell.

[174] Clynes RA, Towers TL, Presta LG, Ravetch JV (2000). Inhibitory Fc receptors modulate in vivo cytotoxicity against tumor targets. Nature medicine.

[175] Kratochvill F, Neale G, Haverkamp JM, Van de Velde LA, Smith AM, Kawauchi D, McEvoy J, Roussel MF, Dyer MA, Qualls JE, Murray PJ (2015). TNF Counterbalances the Emergence of M2 Tumor Macrophages. Cell reports.

[176] Lu M, Sarruf DA, Talukdar S, Sharma S, Li P, Bandyopadhyay G, Nalbandian S, Fan W, Gayen JR, Mahata SK, Webster NJ, Schwartz MW, Olefsky JM (2011). Brain PPAR-gamma promotes obesity and is required for the insulin-sensitizing effect of thiazolidinediones. Nature medicine.

[177] Charo IF (2007). Macrophage polarization and insulin resistance: PPARgamma in control. Cell metabolism.

[178] Miselis NR, Wu ZJ, Van Rooijen N, Kane AB (2008). Targeting tumor-associated macrophages in an orthotopic murine model of diffuse malignant mesothelioma. Molecular cancer therapeutics.

[179] Fujita E, Shimizu A, Masuda Y, Kuwahara N, Arai T, Nagasaka S, Aki K, Mii A, Natori Y, Iino Y, Katayama Y, Fukuda Y (2010). Statin attenuates experimental anti-glomerular basement membrane glomerulonephritis together with the augmentation of alternatively activated macrophages. The American journal of pathology.

[180] Germano G, Frapolli R, Simone M, Tavecchio M, Erba E, Pesce S, Pasqualini F, Grosso F, Sanfilippo R, Casali PG, Gronchi A, Virdis E, Tarantino E, Pilotti S, Greco A, Nebuloni M (2010). Antitumor and anti-inflammatory effects of trabectedin on human myxoid liposarcoma cells. Cancer research.

[181] FOLR2 folate receptor 2 (fetal) http://www.ncbi.nlm.nih.gov/gene?Db=gene&Cmd=ShowDetailView&TermToSearch=2350.

[182] Turk MJ, Waters DJ, Low PS (2004). Folate-conjugated liposomes preferentially target macrophages associated with ovarian carcinoma. Cancer letters.

[183] Nagayoshi R, Nagai T, Matsushita K, Sato K, Sunahara N, Matsuda T, Nakamura T, Komiya S, Onda M, Matsuyama T (2005). Effectiveness of anti-folate receptor beta antibody conjugated with truncated Pseudomonas exotoxin in the targeting of rheumatoid arthritis synovial macrophages. Arthritis and rheumatism.

[184] Puig-Kroger A, Sierra-Filardi E, Dominguez-Soto A, Samaniego R, Corcuera MT, Gomez-Aguado F, Ratnam M, Sanchez-Mateos P, Corbi AL (2009). Folate receptor beta is expressed by tumor-associated macrophages and constitutes a marker for M2 anti-inflammatory/regulatory macrophages. Cancer research.

[185] Nagai T, Tanaka M, Tsuneyoshi Y, Xu B, Michie SA, Hasui K, Hirano H, Arita K, Matsuyama T (2009). Targeting tumor-associated macrophages in an experimental glioma model with a recombinant immunotoxin to folate receptor beta. Cancer immunology, immunotherapy.

[186] Shen J, Putt KS, Visscher DW, Murphy L, Cohen C, Singhal S, Sandusky G, Feng Y, Dimitrov DS, Low PS (2015). Assessment of folate receptor-beta expression in human neoplastic tissues. Oncotarget.

[187] Luo Y, Knudson MJ (2010). Mycobacterium bovis bacillus Calmette-Guerin-induced macrophage cytotoxicity against bladder cancer cells. Clinical & developmental immunology.

[188] Weigert A, Sekar D, Brune B (2009). Tumor-associated macrophages as targets for tumor immunotherapy. Immunotherapy.

[189] Vincent J, Mignot G, Chalmin F, Ladoire S, Bruchard M, Chevriaux A, Martin F, Apetoh L, Rebe C, Ghiringhelli F (2010). 5-Fluorouracil selectively kills tumor-associated myeloid-derived suppressor cells resulting in enhanced T cell-dependent antitumor immunity. Cancer research.

[190] Kodumudi KN, Woan K, Gilvary DL, Sahakian E, Wei S, Djeu JY (2010). A novel chemoimmunomodulating property of docetaxel: suppression of myeloid-derived suppressor cells in tumor bearers. Clinical cancer research.

[191] Shiao SL, Ruffell B, DeNardo DG, Faddegon BA, Park CC, Coussens LM (2015). TH2-Polarized CD4(+) T Cells and Macrophages Limit Efficacy of Radiotherapy. Cancer immunology research.

